# Microfluidic Strategies for Extracellular Vesicle Isolation: Towards Clinical Applications

**DOI:** 10.3390/bios13010050

**Published:** 2022-12-29

**Authors:** Alessio Meggiolaro, Valentina Moccia, Paola Brun, Matteo Pierno, Giampaolo Mistura, Valentina Zappulli, Davide Ferraro

**Affiliations:** 1 Department of Physics and Astronomy, University of Padua, Via Marzolo 8, 35131 Padua, Italy; 2 Department of Comparative Biomedicine and Food Science, University of Padua, Viale dell’Università 16, 35020 Legnaro, Italy; 3 Department of Molecular Medicine, University of Padua, Via Gabelli 63, 35121 Padua, Italy

**Keywords:** extracellular vesicles, microfluidics, purification, liquid biopsy, microfabrication, clinics

## Abstract

Extracellular vesicles (EVs) are double-layered lipid membrane vesicles released by cells. Currently, EVs are attracting a lot of attention in the biological and medical fields due to their role as natural carriers of proteins, lipids, and nucleic acids. Thus, they can transport useful genomic information from their parental cell through body fluids, promoting cell-to-cell communication even between different organs. Due to their functionality as cargo carriers and their protein expression, they can play an important role as possible diagnostic and prognostic biomarkers in various types of diseases, e.g., cancers, neurodegenerative, and autoimmune diseases. Today, given the invaluable importance of EVs, there are some pivotal challenges to overcome in terms of their isolation. Conventional methods have some limitations: they are influenced by the starting sample, might present low throughput and low purity, and sometimes a lack of reproducibility, being operator dependent. During the past few years, several microfluidic approaches have been proposed to address these issues. In this review, we summarize the most important microfluidic-based devices for EV isolation, highlighting their advantages and disadvantages compared to existing technology, as well as the current state of the art from the perspective of the use of these devices in clinical applications.

## 1. Introduction

Discoveries in genomics are leading to important outcomes in medicine, improving knowledge of many diseases and leading to the concept of “precision medicine”, which is defined as the tailoring of medical treatment to individual characteristics [1]. For example, after a cancer diagnosis, the first approach is often a surgical biopsy to identify the type of tumor by specific marker expression or by genomic analysis [2]. Unfortunately, the latter is an invasive and time-consuming procedure, may not be representative of the entire tumor, and may cause cancer seeding [3]. To face these issues, much attention has been paid to a less invasive procedure called *liquid biopsy*: body fluids (e.g., blood, urine, saliva) are screened for tracers released by cancer tissues, which can provide more rapid and complete information about the original tumor (e.g., type, stage, progression, etc.) and could be used as prognostic and/or diagnostic tools [4]. The most well-known tracers are circulating tumor cells (CTCs) and circulating tumor DNA (ctDNA). The first are cells that are spontaneously released from the cancer tissue and travel in the patient’s blood [5]. Similarly, ctDNA are nucleic acid fragments presenting specific tumor mutations that are released from cancer cells and travel in body fluids [6].

Another type of *tracer* that has been discovered in recent decades as potentially useful for liquid biopsy are extracellular vesicles (EVs) [7]. These are double-layered phospholipid membrane structures, released by most cell types, which travel in body fluids carrying various biological molecules of the parental cell (i.e., proteins, lipids, and nucleic acids). The biogenesis of EVs is mainly related to two pathways: (i) the direct outward budding of the cell membrane and ii) the inward budding of multivesicular bodies that fuse with the cell surface to then be released. In the former case, EVs are known as ectosomes (or microvesicles (MVs), or microparticles), with a size of 100 nm to 1000 nm, and in the latter case, they are known as small extracellular vesicles (sEVs) or exosomes, with a size ranging between 30 and 200 nm [8,9]. Although initially considered cell debris or cell waste, it is now recognized that EVs play a role in cell-to-cell communication, acting as cargo ships between cells by transporting genetic information [10], and therefore participating in a variety of physiological and pathological processes [11]. Therefore, EVs are perfectly suitable for liquid biopsy and are now considered promising diagnostic, predictive, and prognostic biomarkers for many types of diseases. In fact, unlike CTC and cDNA, EVs can provide a variety of information, e.g., either on cardiovascular [12], autoimmune [13], and neurodegenerative [14] diseases, or on various types of cancer [15]. 

Today, given the invaluable importance of EVs for liquid biopsy, there are some key challenges to overcome regarding their isolation [16]. The current most frequently used approaches, described in Section 2, are based on differential ultracentrifugation (DU), size-exclusion chromatography (SEC), density-gradient separation (DGS), filtration, and immunoaffinity strategies. Additionally, EVs can be collected from various fluids (e.g., cell culture, blood, urine, etc.); thus, the same isolation strategy may present different efficiencies depending on the starting sample [17]. Finally, most of the methods require at least several hours for EV isolation, and thus more rapid isolation protocols are also demanding.

**Figure 1:**
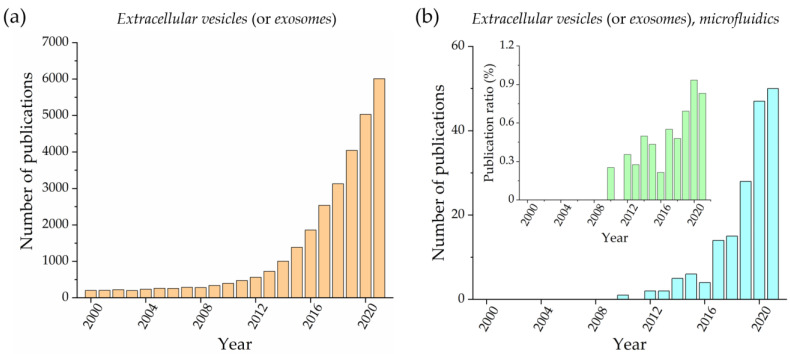
Number of publications per year that contain the keywords: *extracellular vesicles* (or *exosomes*) (**a**) and *extracellular vesicles* (or *exosomes*) and *microfluidics* (**b**). An inset with the ratio between these two numbers is also reported. (Source: Web of Science database, excluding review, meeting abstract, and retracted papers).

Microfluidic devices have recently been proposed for addressing these issues, as demonstrated by the increasing number of published papers that have appeared over the past ten years on this topic. Figure 1 compares the publications per year obtained using the terms *extracellular vesicles *(or *exosomes*) (Figure 1a) and together with *microfluidics* (Figure 1b) as keywords. Both trends are similarly increasing; however, the ratio between the two numbers (Figure 1b, inset) reports how microfluidics has gained slightly more visibility during the last five years. Notably, considering the low number of articles per year in the microfluidic case, this trend must be monitored in the near future. Microfluidics is commonly defined as the science and technology of systems that manipulate small amounts of fluids (pL and nL ranges), using channels with dimensions that typically range from tens to hundreds of microns [18]. This leads to several advantages, including the development of a portable system for point-of-care analysis, the reduction in sample and reagent volume, down to a million times more than conventional approaches, and the ability to perform parallelized assays that can drastically increase analysis throughput [19]. Given these benefits, it is clear that microfluidics can contribute to simplifying and speeding up the EV isolation process from biofluids, representing a good alternative to conventional protocols. Additionally, microfluidic devices can also be exploited for EV analysis and detection, being embedded within the same microfluidic system or based on other instruments (e.g., a fluorescence microscope). In the latter case, microfluidic devices can be seen as passive tools for EV storage.

**Figure 2:**
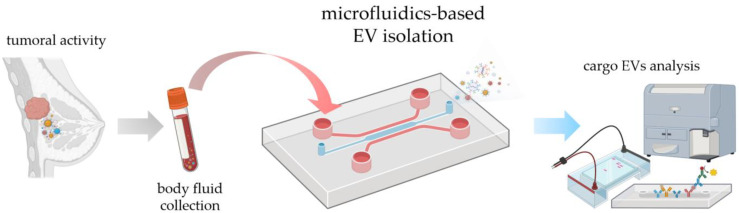
Possible workflow of tumor diagnosis using the microfluidic EV isolation strategy, from sample collection by liquid biopsy to analysis. The elements of the figure are created by BioRender.com.

In this review, we aim to address the most relevant microfluidic systems devoted to EV isolation, underlining both advantages and disadvantages compared to the conventional existing methods. After reviewing the most common isolation methods, microfluidic approaches are discussed, with particular emphasis on those that seem more promising for future clinical applications. In this context, EVs must be isolated by a microfluidic device and ready for further analysis (see the workflow in Figure 2). Given the variety of microfluidic devices in terms of microfabrication and functionality, they are divided into two main categories: physical and chemical approaches. Whereas the former can be distinguished in active and passive methods, the latter are mainly based on immunocapture on fixed and non-fixed (beads) substrates. In addition, a quantitative analysis of the diffusion of the various microfluidic methods, as well as their capabilities of being used in real clinical studies, is presented. A short description of EV detection methods based on microfluidics is also introduced; however, for a deeper understanding, a dedicated review can already be found in the literature [20].

Importantly, in recent years, different terminology has been used in the literature to classify EVs based on their size or function, since their biogenesis was not easily assessed; however, in 2018, the International Society for Extracellular Vesicles (ISEV) indicated using the word *Extracellular Vesicle* (or EV) as a broad generic term that includes all subtypes of vesicles to avoid confusion in the literature. This recommendation is followed in this review, using the term small extracellular vesicles (sEVs) when the cited articles refer to exosomes or EVs smaller than <200 nm [9].

## 2. Conventional EV Isolation Strategies

Conventional methods for the isolation and purification of extracellular vesicles can be classified into methods based on the morphological properties (i.e., size, density) and based on their interaction with specific components (solubility, protein reaction). The preferential strategy must be chosen as a function of the initial sample (e.g., cell culture, blood, urine) and the scope of the analysis (e.g., quantity evaluation, diagnosis of specific diseases) [9]. In the following paragraphs, conventional methods will be briefly introduced, reporting their working principle and focusing on their advantages and disadvantages in terms of EV purification. More details on these methods can be found in a specific review [17].

### 2.1. Differential Ultracentrifugation and Density Gradient Ultracentrifugation

Differential ultracentrifugation (DU) was the first approach used for EV isolation. In general, centrifugation is a label-free method that allows accelerating the natural sedimentation rate of suspended objects that are denser than the surrounding medium [21]. In the case of EV isolation, protocols are typically based on increasing the centrifugal force to progressively remove first cell debris (approximately 1500× *g*), then large EVs (between 10,000 and 20,000× *g*), and finally, to collect small EVs (100,000–200,000× *g*) appearing as a small pellet at the bottom of the centrifugal tube [21]. The limitations of this method are related to the need for expensive equipment (i.e., an ultracentrifuge) and the variable recovery rate (between 5 and 80%), which can often be operator related, preventing comparisons between different studies. In addition, isolated objects are pelleted according to their density, and thus collected materials also contain protein complexes and non-EV nanoparticles (e.g., apoptotic bodies, viruses), leading to an incomplete separation [22]. Additionally, EVs may also cauterize together due to strong centrifugal force. Many different DU protocols can be found in the literature that may puzzle those who are in the research field for the first time. Despite these issues, probably due to its simplicity and recovery rates that can be rather high, DU remains the most widely used approach in research laboratories and is typically combined with other filtration-based EV isolation methods. 

Density gradient ultracentrifugation (DGU) is a variation of DU that consists of the addition of specific components (e.g., sucrose) within the suspending medium in order to match the EV density, while allowing the other components to precipitate [23]. Although DGU allows for gaining higher EV purity than DU, some limitations are also reported: the process is time consuming, and molecules with similar EV density (e.g., high-density lipoproteins) can be co-isolated.

### 2.2. Filtration Methods (Ultrafiltration and Size-Exclusion Chromatography)

Filtration methods are based on the use of a porous membrane to filter objects larger than the porous size [24]. Since EVs typically have sub-micrometric size, membranes with pore sizes between 0.001 µm and 0.01 µm are used combined with ultracentrifugation, in the so-called ultrafiltration (UF) technique; UF allows for faster protocols and better sample quality than DU in terms of purity from protein co-isolation. However, the recovery yield can be biased by the pore size [25].

A highly used filtration-based technique for EV isolation is size exclusion chromatography (SEC), consisting of the elution of EVs in a column composed of packed porous polymeric beads [26,27]. This simple strategy allows for isolating intact EVs from various biological fluids, preserving their biophysical properties, sharp-peaked distribution in size, and high functionality. Thus, among isolation strategies based on the physical properties of EVs, in particular their size, SEC is considered the least invasive in terms of EV integrity [28]. On the other hand, an important disadvantage is the low recovery rate: SEC can be applied to concentrate EV fluids (such as plasma), but in the case of low initial EV concentration (e.g., cell culture media), a pre-concentration step by UF is required. Despite the similarity to microfluidic technologies based on separation by size through pores, SEC relies on the passive motion of particles in a stationary phase, and it is highly time-consuming. Importantly, SEC can also co-isolate other components, such as viruses, protein aggregates, large proteins, and low-density lipoproteins; however, these contaminations are typically less compared to other EV-isolation methods. Today, several commercially available SEC kits can be found specifically designed for EV isolation, depending on the volume and quality of the input sample [29]. It should also be noted that recent studies are trying to improve the capabilities of a standard SEC column, for example, by using a bead size gradient as in particle purification liquid chromatography (PPLC) [30,31]. In any case, despite the good purity of the final sample, SEC requires high costs for disposable filtering columns, in addition to long isolation times.

### 2.3. Precipitation and Immunoaffinity Methods

Physical–chemical interactions between EVs and solid support are also exploited for their isolation. These approaches are based on EV precipitation [32], adjusting their solubility by chemical compounds, and immunochemistry reactions that exploit the protein present on their surface [33]. EV precipitation can be achieved by properly adjusting the concentration of specific polymers within the starting sample, leading to very simple protocols (e.g., ExoQuick^®^ [34]). However, the final samples are contaminated by the polymer used, which may compromise the downstream analysis. In contrast, immunochemistry reactions are based on the chemical binding between proteins on the EV membrane and specific antibodies, typically grafted onto surfaces or beads. In more detail, some specific tetraspanin molecules are present on most EV membranes (e.g., CD63, CD81, CD9, and others) and are typically used for this purpose [35]. It is noteworthy that the same approach can be applied to isolate a subpopulation of EVs that presents specific membrane proteins associated with a specific EV subtype. The advantages of this approach are its simplicity, the fact that it does not require specific training by the user, and its reproducibility. However, to avoid nonspecific interaction, pre-purification steps are typically required (e.g., differential ultracentrifugation). In addition, isolation kits for specific immunocapturing are usually costly. Another drawback is intrinsic in the approach itself: by selecting the EVs from their surface markers, a subpopulation is always collected, and this could eventually bias the downstream analysis. For this reason, it is preferable to use multimarker antibody cocktails to recover vesicles characterized by different antigens or secreted from heterogeneous cells [36,37]. 

### 2.4. Comments

In summary, it is clear that all the conventional approaches listed above have both advantages and disadvantages, as highlighted in Table 1, and the choice between them must be made according to the scope of the study. However, it is important to note that although the chosen approach is the same, the specific parameters for the isolation of EVs are adapted differently from time to time in different laboratories, leading to a lack of standardization and important inconveniencies in comparing the data. On the contrary, microfluidic devices have the potential to overcome some issues, such as the need for expensive facilities and consumables and large sample volumes, which are peculiar to conventional isolation techniques.

## 3. EV Isolation Methods Based on Microfluidic Devices

Microfluidics is typically applied to bioanalytical protocols by following two different approaches: (i) the miniaturization of existing methods or (ii) the development of new methods that cannot be performed without miniaturization. The first way allows for automating processes while reducing starting volumes, sometimes leading to increased throughput of the analysis and quality of the output sample. In contrast, the second approach tackles the conventional limitations from a completely different angle, leading to results that cannot be compared with existing large-scale methods.

Among the possible ways to classify microfluidic devices, they can be distinguished between “physical” or “chemical” methods according to the nature of forces that regulate the EV isolation process.

### 3.1. Physical Methods

Physical isolation methods are label-free and exploit physical properties to discriminate vesicles (i.e.,: size or density). They can respond to external physical forces or be based on passive EV collection. In the following, physical approaches are divided into passive or active methods, depending on the presence or absence of driving forces that trigger the physical characteristics of the EVs or of the medium in which they are dispersed. 

#### 3.1.1. Passive Approaches

Passive separation methods are label-free isolation strategies that do not require external forces or stimuli. They are intended to enrich EVs by filtering processes through membranes integrated in microfluidic channels or by exploiting hydrodynamic flow properties.

**Figure 3:**
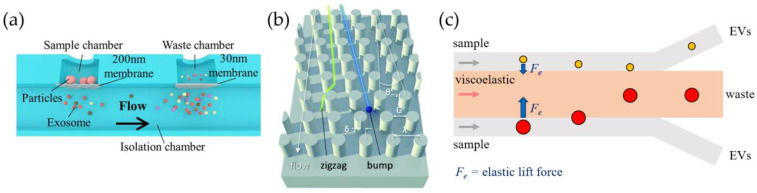
EV isolation methods based on passive approaches. (**a**) Double-membrane system to enrich sEVs (exosomes) of sizes between 30 and 200 nm [38]; (**b**) silicon array of pillars placed inside the chip to induce different zigzag pathways based on particle dimension [46]; (**c**) microfluidic device allowing collection of extracellular vesicles from initial sample mediated by elastic force from viscoelastic fluid.

Filtration. A simple method to separate EVs from the initial biological sample based on size requires the use of filtration systems, such as a nanoporous membrane, that allow the passage of vesicles having a dimension smaller than the pore size by exploiting pressure provided by external syringe pumps or by pressure controllers. Inspired by UF and SEC, microfluidic protocols have important advantages in terms of cost, required sample volume, and automation. Filters such as polycarbonate track-etched membranes were integrated within microfluidic devices during the fabrication process by the authors of [38,39,40,41,42,43,44]. In this way, the final device is simple to use: it does not require labels or surface treatments, allowing for the processing of very large sample volumes. Nevertheless, the production of such devices can be very complicated because of the strong microfabrication skills required, sometimes preventing mass production. As in the case of conventional filters, these devices can be prone to clogging and are typically disposable; additionally, isolation through filters lacks specificity, except for size. Liang et al. presented a prototyping example, developing a polycarbonate-based double-filtration system to isolate vesicles within a range of 30–200 nm starting from the urine of patients with bladder cancer [38]. An isolation chamber is devoted to collect EVs smaller than the pore size of 200 nm of the first membrane, and particles smaller than 30 nm are trapped through a second filter in a waste chamber (Figure 3a). This method allowed for the isolation of EVs from 8 mL of the pre-centrifuged initial sample in approximately 3.5 h, using a flow rate of 40 µL/min. Further multiple-step filters are arranged in the Exosome Total Isolation Chip (ExoTIC), in which five membranes in a series (pores of 200, 100, 80, 50, and 30 nm) allow for a strict differentiation in the size of vesicles from various types of samples, such as plasma, urine, and lavage [39]. The starting samples have an upper volume limit that can range from 20 mL for cell culture to 500 µL for plasma. Another device called Exodisc takes advantage of centrifugal force with double-step filtering through membranes of different pore sizes [44]. Woo et al. were able to isolate exosome from a 1 mL starting sample of cell culture or urine in 30 min with high purity (95% yield). Sunkara et al. optimized the same platform for processing whole blood, despite using smaller samples (30 µL) and reaching lower recovery rates (exceeding 75%) [45]. Other types of filtering devices rely on cross-flow (tangential-flow) processes, in which the feed flows across the surface of the membrane that acts to concentrate EVs larger than the pore size (30–50 nm). These devices can pair the cross-flow strategy with conventional (dead-end) filtration [41,42] or with other isolation techniques, such as immunoaffinity-based capture [43], to improve purification efficiency (see also Section 3.2.1).

Inertial force. EV separation can also be performed by exploiting the inertial lift force $F_{i}$ that particles experience while flowing in a microchannel due to the Poiseuille flow profile [47,48,49]. In fact, $F_{i}$ acts to drive the particles orthogonally to the flow direction inside the microchannel in a manner that strongly depends on particle dimension D ($F_{i}\;\propto \;D^{4}$). Therefore, by properly tuning the channel size and flow rates, it is necessary to focus the particles according to their size [50,51,52,53,54,55]. A particular configuration consists of spiral channels that strongly favor the lateral migration of particles with different velocities depending on size [56], as used by Tay et al. for rapid isolation from a whole blood sample at 80 µL/min, despite reaching a poor recovery efficiency (20–60%) [57].

Deterministic lateral displacement (DLD). A different approach exploits inertial microfluidics and hydrodynamic interactions of particles with structured channels: particles flowing in microchannels, other than the main force that drives them along the channel itself, also experience lateral forces depending on their size. This effect can be combined with specific and ordered patterns of pillars inside microfluidic channels (see Figure 3b), leading to the so-called deterministic lateral displacement (DLD), which allows for the generation of streamlines that the particles follow depending on the distance of centers λ and the gap of the pillars G, as well as on the offset angle θ. Their separation can occur whenever the particle size exceeds a critical diameter, which for circular pillars equals $D_{C}=1.4\;G\tan\theta^{0.48}$, which acts as a cut-off [58]. Specific details about the physical principles of DLD are discussed in devoted articles [59]. DLD has been widely applied for hydrodynamic cell separation depending on the cells’ size, and it has also recently been applied to nanometric objects, such as EVs. For example, the pioneering work of Wunsch et al. provided a sharp resolution of particles between 20 and 110 nm separated with a silicon-based pillar array [60]. The production of these pillars, having gaps from 25 to 235 nm, required a sequence of complex micro- and nano-fabrication steps including photolithography followed by reactive-ion etching, then an electron beam process, and finally, deep-UV lithography. Later, Smith et al. integrated 1024 arrays in parallel in another nanoDLD device (Figure 3b) to isolate EVs from serum and plasma, using a similar fabrication approach [46]. This improved design allows for faster isolation processing (up to 900 µL/h), despite an EV recovery of approximately 50%. Other devices, reporting pillar dimensions and gaps of the order of microns, are instead replicated from silicon wafers produced by standard photolithographic and etching techniques, allowing one to reach a high purity of the final sample, but working at low throughput (of the order of µL/h) [61,62].

Viscoelastic force. Most bodily fluids (such as blood, saliva, semen, etc.) exhibit a non-Newtonian behavior when flowing through channels [63]. This viscoelastic property can be leveraged to separate particles by size by driving a lateral migration owing to the elastic lift forces, without external fields. Specifically, the trajectory of the particles is regulated by the first normal stress difference ($N_{1}$), inducing the lateral motion towards the points of minimum shear rate, with relaxation time dependent on medium properties and channel width [64,65,66]. The resulting elastic lift force $F_{e}$ depends on the cube of the particle size ($F_{e}\;\sim \;D^{3}$), and therefore, taking into account a device presenting several outlets (see Figure 3c), larger particles migrate faster to the center line of the channel, whereas smaller EVs are collected at the two sides [67,68,69]. Unlike DLD-based devices, particles immersed in viscoelastic media can be focused simply by adjusting the rate and width of microchannels, without requiring additional micro- or nanofabricated structures [67,68]. However, to enhance the elastic effect and guarantee good hydrodynamic focusing, specific polymers can be added to the starting samples. As examples, Liu et al. (2017) added a low concentration (0.1 wt%) of a biocompatible polymer to the cell culture medium or serum sample, namely, poly(oxyethylene) (or PEO), to enhance these effects and better control the separation of EVs, achieving high purity and recovery rates greater than 80% and 90%, respectively [70]. Then, in 2019, a similar approach was used to simultaneously separate particles by size and based on membrane protein EVs from breast cancer cell lines and from serum, by using double-stranded λ-DNA molecules in TBE buffer to increase the non-Newtonian effect [71]. In this case, the extracellular vesicles are subjected to the centerline-directed elastic lift force $F_{e}$; additionally, larger microvesicles and apoptotic bodies are repelled by the elastic force, competing with the and drag forces $F_{d}$ ($F_{d}\;\propto \;D$). Asghari et al. exploited oscillatory flows to separate micrometer and sub-micrometer constituents from HEK293T cell lines and was able to focus both λ-DNA strands and vesicles in a sheathless flow [72]. For this purpose, a more complex setup is needed to perform the EV separation, including a pressure-driven chip coupled with an electronic device to actuate valves and generate controlled flow oscillations. 

Flow fractionation methods. Another possible way to separate microparticles by size by exploiting hydrodynamic forces is provided by asymmetric flow field-flow fractionation (AF4) [73]. This method requires the implementation of thin microchannels (dozens of µm) having one side made of a membrane that allows the generation of a flow perpendicular to the main stream [74]. Thus, the injected sample under laminar flow conditions is subjected to both the cross-flow field and Brownian diffusion. The accumulation of particles is regulated by the competition of these two counteracting forces, which induce large particles to move in proximity to the membrane, and the smaller particles are easily conveyed along the stream. Typically applied for polymer and protein fractionation, AF4 has been used to isolate EVs, being capable of separating two different subpopulations of vesicles by size (60–80 nm and 90–120 nm) from several tumor cell lines [75]. Shin et al. employed a similar fractionation approach, known as EV separation pinched-flow fractionation (PFF) [76], to isolate EVs from apoptotic bodies [77]. Here, a sheath fluid is applied to achieve EVs by focusing within the microchannel.

#### 3.1.2. Active Approaches

Whenever physical forces are applied to fluids that contain suspended particles, they can respond to the stimulus by changing their motion. Active separation exploits applied fields, such as acoustic or electrical ones, without resorting to channel functionalization or patterning, being label-free and contact-free.

**Figure 4:**
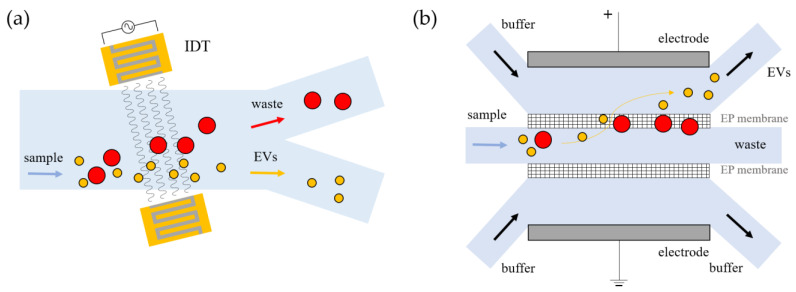
Examples of physical forces to separate vesicles within microfluidic chips. (**a**) Device with interdigitated electrodes generating acoustic waves to separate EVs from the initial sample; (**b**) differentiation of EVs through membranes mediated by electrophoretic (EP) forces.

Acoustofluidics. Acoustofluidic devices combine the ability of microfluidics to handle small volumes in confined channels with the ability to trigger particle motion with acoustic waves [78,79,80]. This label-free and contact-free method employs ultrasound waves to induce differential forces on particles according to their size [81]. Particles can be trapped, separated, focused, or transported by regulating the properties of acoustic waves, which can propagate within the bulk material (bulk acoustic waves, BAWs) [82,83] or along the surface of the medium (surface acoustic waves, SAWs) [84]. Importantly, to emit acoustic waves, electrodes or piezoelectric substrates must be included into the microfluidic devices and properly engineered during their production. In the case of BAWs, the entire piezoelectric material driven by an alternating current (AC) vibrates at the same frequency of the AC signal (100 kHz–10 MHz). In contrast, SAWs are generated by applying an AC signal to interdigitated transducers (IDTs) patterned on a piezoelectric material, which are excited at higher frequencies than BAWs (up to GHz) (Figure 4a). In order to confine small particles such as EVs, high frequency, of the order of dozens of MHz, is generally required, and thus SAW-based devices are employed [85]. As an example, Lee et al. used a LiNbO_3_ wafer to imprint interdigitated electrodes that can discriminate sEVs and larger microvesicles from red blood cells according to their size; their cutoff value can be set by tuning the acoustic power and flow velocity [86]. Although fabrication is somewhat complex due to the presence of acoustic actuators, acoustofluidic devices can guarantee high isolation efficiency [87,88,89]. Wu et al. developed a device based on SAWs consisting of two modules to remove larger blood cells and debris, showing separation of EVs with an 82.4% recovery rate and 98.4% purity using flow rates in the order of few µL/min [90]. Other SAW-based platforms have been coupled with commercial acoustic transducers that lead to automated processes [91,92,93], and others have been implemented together with modules devoted to the detection of sEVs [94,95,96].

Electrokinetic force. Electric fields applied to fluids allow for the manipulation of polarizable particles, giving rise to a variety of electrokinetic phenomena: electrophoresis, dielectrophoresis, electroosmosis, etc. These effects provide forces whose magnitude acting on the particles is strictly dependent on their dimension, the dielectric constant, and the charge density of both particles and the surrounding medium. More precisely, the electrophoretic effect (EP) works on monopoles, requiring high forces ($F_{EP}\;\propto \;E$) to induce particle manipulation. Dielectrophoresis (DEP) instead allows for controlling the trapping of particles by electric field gradients, to which the DEP force is proportional ($F_{DEP}\;\propto \;\nabla {\left|E\right|}^{2}$) and thus depends on the electrode geometry, rather than the intensity of electric pulses [97]. The latter method happens to be the most widely employed for EV manipulation due to its simplicity with respect to the other electrokinetic phenomena. Ibsen et al. used an alternate current electrokinetic microelectrode to concentrate sEVs from plasma on the edge of the microelectrodes where high field gradients were exerted to process relatively small aliquots (30–50 µL) in less than 30 min, including on-chip fluorescent detection [98]. To improve isolation performance, a device based on dielectrophoretic interactions can be mediated by other types of substrates, such as polystyrene microspheres [99], coupled with automated parts [100], or pneumatically driven components [101]. The latter has been exploited by Davies et al. in a device that takes advantage of the electrophoretic interaction with a pressure-driven filtration stage of porous polymer monolithic membranes (PPMs), which have variable size pores, to isolate vesicles from 240 µL of whole blood in two hours. In addition to DEP, other examples of electrokinetic phenomena already exploited to trap and concentrate vesicles are electrophoresis [102,103] or electro-osmosis [104,105]. For example, Cho et al. developed a device to enrich plasma EVs by coupling a porous membrane with a dedicated electrode (Figure 4b), in order to remove free proteins and debris subjected to electrophoretic migration through 30 nm pores, with an efficiency of approximately 85% [106]. 

A recent work by Tayebi et al. combined both dielectrophoretic and acoustophoretic forces to sort extracellular vesicles (<200 nm) and microvesicles (>300 nm) from cell cultures that reached high levels of purity (95%) and recovery (81%) [107]. This kind of virtual DLD (vDLD) permits tuning the balance of the two counteracting forces by adjusting properties of the medium and channel sizes, given a fixed electrode geometry.

### 3.2. Chemical Methods

Unlike physical methods, approaches based on the chemical affinity between specific antibodies and antigens allow for the recovery of vesicles in a more selective manner. Immunoaffinity-based capture can occur on flat or patterned substrates, as well as on micrometric solid beads or nanoparticles. 

#### 3.2.1. Immunocapture on Fix Support

The selective separation of EVs can be achieved by properly engineering the internal microchannel surfaces by adding a specific antibody that can anchor a specific EV membrane protein. The method provides an extremely good specificity and reproducibility and, in the best cases, allows for processing of samples with very high throughput, even of tens of µL/min.

**Figure 5:**
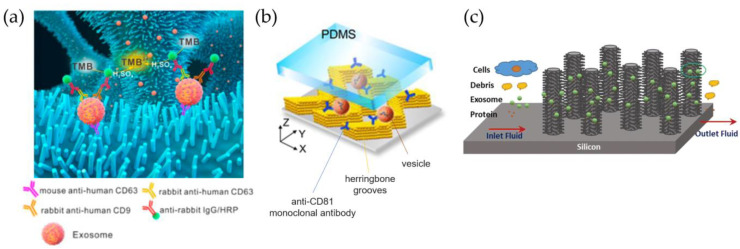
Immunoaffinity capture inside microfluidic devices. (**a**) ZnO nanowires fabricated inside channels for the specific capture of CD63-positive sEVs, modified from [109]; (**b**) colloidal structures arranged in a herringbone configuration inside the microfluidic channel, modified from [123]; (**c**) ciliated silicon nanorods capable of discriminating sEVs from cell debris [113].

The simplest strategy is to functionalize unstructured channels. However, by using a straight channel with a typical lateral side of dozen to hundreds of μm and considering the typical size of EVs, the binding area available for vesicle capture is relatively low, causing a poor probability of contact. Moreover, the laminar flow prevents the correct mixing of the solution containing EVs, limiting their accessibility to the molecules anchored on the channel walls. This issue has been faced in two ways: i) improving mixing by patterning channels with specific patterns and ii) including micro- and nanostructures within the channel by creating a sort of filter through which the solution is forced to pass. The first method is well known in the microfluidic community, having already been applied to promote chemical reactions or for isolation purposes [108]. In contrast, the second mimics standard filtration methods by integrating specificity, since these ‘filters’ are coated to capture EVs showing the desired markers [109]. However, as for filtration methods, this approach suffers from clogging and highly complex microfabrication protocols. To increase the surface-to-volume ratio of channel walls, the inner surfaces of microfluidic chips with micro- and nano- structures are also chemically functionalized with antibodies to ensure the EV chemical affinity [110,111]. The most common microstructures are ordered rows of pillars [112,113,114], herringbone patterns [108,111,115,116,117,118], and properly shaped microposts [119,120]. Meanwhile, in the case of nanopatterning, nanorods, nanowires, and more complex 3D structures [121] are typically used. In 2010, a pioneering work by Chen et al. described a way in which to modify PDMS microfluidic channels presenting herringbone grooves with specific surface treatment [122]. The authors flushed inside the chip a solution of 3-mercaptopropyl trimethoxysilane and incubated it with Neutravidin solution before functionalizing it with biotinylated anti-CD63 antibodies, allowing for the isolation of vesicles from 400 µL of serum within one hour. In another work, Chen et al. used an array of ZnO nanowires (Figure 5a) with interconnected macropores to expand the trapping area [109]. The latter approach was validated with small EVs spiked in saline solutions, showing a trapping rate of up to 30 µL/min, and then with both serum and plasma, from which trapped vesicles were detected with horseradish peroxidase (HRP)-labeled antibody to allow for colorimetric sensing using 3,3′,5,5′-tetramethylbenzidine (TMB). Zhang et al. developed a chip with graphene oxide/polydopamine (GO-PDA) interfaces that provides specific EV absorption from human plasma [119]. The same group then compared a herringbone pattern with solid structures with colloidal silica nanorods (Figure 5b) that showed an improvement in the detection limit of plasma samples at 10 sEVs/µL [123]. Wang et al. fabricated a microfluidic chip structured with a 3D array of ciliated silicon pillars for multiscale filtering of EV-like vesicles or liposomes, mixing filtration properties and immunocapture [112]. Tests with prototyped 83 nm liposomes revealed a retention rate of approximately 60% from a 30 µL of starting volume. This arrangement has been optimized by Qi et al. to improve capture efficiency and preserve EV integrity for drug delivery (Figure 5c) [113]. In this work, the retention rate of sEVs from MDA-MB-231 (breast cancer) cell culture could be increased to 70%, mainly due to the anti-CD63 functionalization of micropillars.

#### 3.2.2. Immunocapture on Beads and Nanoparticles

Another strategy to chemically trap EVs requires the use of beads of a size between 0.5 and 20 μm functionalized with the target antibody. In fact, floating beads of micrometer size present a larger surface area. Beads can be directly injected and mixed within the initial sample, enhancing the EV contact probability, without introducing complicated micro- or nano-structures into the microfluidic chip, which typically require costly fabrication approaches and a highly trained operator. Therefore, this technique is one of the most efficient in terms of specificity, but especially for higher recovery rates and analysis sensitivity. On the other hand, the flow rates applied in the microchannel to transport liquids cannot be too low, in order to prevent bead sedimentation, nor too high, to ensure good mixing between vesicles and EVs, even though some works tried to process the sample at a throughput of up to approximately 9 mL/h [124]. There are different possible beads that can vary in terms of material or size. The most commonly used are micrometer-sized commercial immunomagnetic beads that have a paramagnetic core that can be easily handled using external magnets [125,126,127,128,129,130,131,132,133,134,135]. Here, unlike in functionalized channels for immunocapture, an external magnetic force must be applied to manipulate particles and favor isolation. A key aspect in the choice and use of the floating substrate for EV capture is that the time must be sufficient for the substrate to settle in the channel. The sedimentation speed v for a single object dispersed in a viscous fluid can be calculated by the Stokes law: $v=\left(d^{2}\left(\rho_{d}-\rho_{f}\right)g\right)/\left(18\mu_{f}\right)$, where d and $\rho_{d}$ are the diameter and the density of the object, $\rho_{f}$ and $\mu_{f}$ are the density and the viscosity of the fluid, and g is the gravity acceleration [136]. Thus, considering a polystyrene bead of 1 µm (ρ~1.05 g/cm^3^) and an EV of 200 nm (ρ~1.1 g/cm^3^) dispersed in water ($\mu_{f}$~1 cP), it is possible to notice that the first sediments are more than ten times faster than the second. This occurrence becomes even more critical when using magnetic beads (ρ~1.8 g/cm^3^), as their sedimentation speed is two orders of magnitude higher than for EVs. Therefore, the flow rates applied to the liquid within the channel must be fast enough to prevent particle sedimentation, but slow enough to ensure a sufficiently long incubation time for EV capture. Thus, the working range of this type of device is limited. 

**Figure 6:**
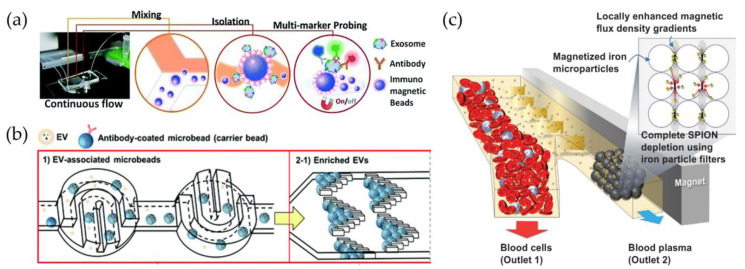
Functionalized beads or nanoparticles used for vesicle capture inside microfluidic devices. (**a**) Immunomagnetic beads coated for the enrichment of vesicles from blood plasma inside ExoSearch chips [126]; (**b**) streptavidin-coated polystyrene beads used as substrate to trap vesicles in herringbone filters and redisperse them [137]; (**c**) microfluidic systems used to flow blood samples and isolate vesicles by means of superparamagnetic nanoparticles (SPIONs), modified from [138].

A highly cited example is given by He et al., who used immunomagnetic beads coated with specific antibodies (e.g., anti-EpCAM) to capture and lyse sEVs inside a unique device to analyze the protein content by chemifluorescent ELISA [125]. Plasma samples of 30 µL volume are processed in about 100 min. Zhao et al. fabricated a device called ExoSearch to enrich sEVs from plasma and measure multiple marker fluorescent signals (Figure 6a) [126]. 

In addition to magnetic beads, polystyrene beads (PS) can be used for the isolation of EVs [80,99,124,137,139,140,141] using centrifugation and redispersion instead of magnetic forces. One of the main drawbacks of immunocapturing with beads is the difficulty of breaking the bond with antibodies, preventing EV damage. However, Tayebi et al. used specifically coated PS beads to capture EVs from MCF-7 (a breast cancer cell line) in constrictions along the microfluidic circuit with an aperture of 30 μm to trap a single bead, by exploiting hydrodynamic resistance in channels having different shapes [140]. The trapped EVs were then removed from the beads by rising with a low pH IgG elution buffer (0.1 M glycine-HCl) for 1 min and then waiting 10 min for antibody–antigen dissociation. Finally, neutralization is provided by a solution of pH 7.4 (1 M Tris-HCl). Despite a good purity, this approach limits the amount of EV–bead complexes to the number of trapping sites and the maximum flow rate (50 μL/min). Gwak et al., instead, promoted chaotic stirring of coated PS beads inside horseshoe-shaped mixers, and the work [124], was able to enrich plasma EVs using fish trap-shaped filters (Figure 6b) [137]. Then, using the same elution buffer, different flow rates were tested: under the best conditions, the whole isolation process for the 100 μL sample was completed in 5 min, showing a capture efficiency greater than 97%.

Unlike micrometric beads that are typically larger in size than EVs, an alternative solid substrate is represented by nanoparticles (NPs), which are on the order of a few nanometers, either with magnetic properties (superparamagnetic or ferromagnetic) [134,138,142,143,144,145,146,147] or simply functionalized with selective markers [148]. In particular, they have a similar or even smaller size than small EVs, so EVs can be used to capture a single EV on their surface, rather than being encapsulated inside, as used in drug delivery [149]. Notably, these NPs can be an active tool for the capture and manipulation of vesicles, rather than a passive substrate. A pivotal work by Shao et al. showed a strategy for labeling and isolating blood glioblastoma microvesicles, implementing properly functionalized magnetic nanoparticles (core of 7 nm) and using a two-step protein targeting to maximize binding. This approach allowed for better detection of CD63 + vesicles with a micronuclear magnetic resonance (µNMR) system [142]. In another work, Ko et al. exploited magnetic NiFe nanopores (600 nm diameter) to trap EVs labeled with 50 nm coated NPs, allowing for the processing of serum and plasma with flow rates of up to 10 mL/h [144]. Recently, increasing attention has also been paid to superparamagnetic iron oxide nanoparticles (SPIONs) to isolate EVs due to the nanoparticles’ reversible magnetic property and easy manipulation, as exploited by Kwon et al. for the purification of blood samples [138]. Here, SPIONs and EVs create a complex cluster that can be isolated by exploiting the magnetic force applied by an external magnet (Figure 6c).

In the following, Table 2 reports a summary of the main EV isolation methods exploiting microfluidic strategies, distinguished between physical and chemical, together with their working principle.

## 4. Discussion

### 4.1. Physical and Chemical Microfluidic Approaches: Pros and Cons

When microfluidic devices are designed to improve the purification and/or isolation of specific analytes from complex starting samples, four main aspects must be considered to balance advantages and disadvantages: throughput of the process, quantity and quality of the output sample, automation capability, and complexity of the microfabrication. In the following section, we discuss the above-presented approaches for EV isolation in microfluidic devices, focusing on these four aspects. A schematic summary of the following discussion is also reported in Table 3. 

Physical approaches based on filtration, although based on principles similar to those of UF and SEC, allow for the processing of large volumes at high throughput. In fact, microfluidic protocols can be easily automated with the support of external pumps controlled by devoted software [189] to infuse biological samples at high rates throughout filters inside channels. However, the realization of these microfilters represents a critical aspect: as for their correspondent conventional methods, these devices are typically disposable, because they are prone to clogging, and the processing time for complete isolation is quite long compared to that of the other microfluidic techniques.

In contrast, physical approaches based on external stimuli, such as acoustic waves or electric fields, are not directly comparable to existing methods. These contact-free strategies prevent EV damage and preserve the EVs’ functional properties [86,106]. In fact, the presented results show good vesicle integrity and a homogeneous size distribution, especially in terms of the acoustic approach [90]. On the other hand, captured EVs have a low purity, because other contaminants that have a similar size and/or density can be isolated together. Then, as in the previous case, the microfabrication requires both an appropriate environment (i.e., a clean room) and trained operators, since the electrodes must be integrated inside microfluidic devices, and high alternate electric fields must be applied and precisely controlled. The latter practice must be executed by equipped laboratories, which include costly facilities, such as photolithographic platforms, metal evaporators, electronic equipment, and related characterization instruments. However, compared with microfluidic filtering methods, cleaning protocols can be considered in the case of SAW or DEP devices, partially reducing the impact of the microfabrication. Most of the aforementioned strategies based on separation by size can be biased by the fact that other membrane-based components also have dimensions similar to those of EVs (e.g., lipoproteins).

Then, approaches rooted in chemical affinity have been integrated within microfluidic devices, with the aim of improving the anchor point by increasing the surface-to-volume ratio, isolation throughput, sample quality output, and process automation [190]. Compared to physical approaches, immunoaffinity provides a highly specific isolation that allows for the distinction between the EV subpopulation [191] and the high purity of the isolated sample. In the fixed support-based approach, molecules are coated on microchannel surfaces that typically present specific micro- and/or nanopatterns to improve the liquid mixing (microstructures) and/or acting as filters (nanostructures). In contrast, the use of micrometric beads as solid floating supports for EV capture allows for using more simple channel geometries, since the beads themselves act as traps that improve the surface-to-volume ratio. However, compared to other microfluidic approaches, the use of micrometric beads leads to difficulties in terms of the complete automation of microfluidic devices, since they are prone to sedimentation and can induce microchannel clogging [144]. Another disadvantage of this approach is the high costs of purchasing commercially available microbead kits devoted to EV capture; therefore, custom and home-made protocols are generally preferable [139]. A completely different perspective is provided using nanoparticles. They present a size comparable to or even smaller than a single EV, leading to an improvement in capture control and preventing damage to the EVs. Additionally, since several characterization methods are now based on optical and spectroscopic techniques (e.g.,: fluorescence microscopy, Raman spectroscopy), nanoparticles can be directly used to improve the signal-to-noise ratio [188]. A possible drawback of the use of NPs is that they require trained operators for their synthesis and, importantly, their stability is strongly influenced by the surrounding buffer, which may affect the EVs as well. Therefore, strong physical-chemical expertise is required to develop a working microfluidic device based on NPs’ isolation.

In general, as summarized in Table 3, among the microfluidic approaches analyzed, we considered devices based on the chemical affinity of the fixed support to be the most promising, as they show good throughput and high-quality sample output, supported by simple microfabrication and automation. In this sense, the use of beads requires more precautions due to the possible sedimentation and clogging issues. In contrast, physical approaches suffer from complicated complex microfabrication requiring electrode integration, which, at the current state of the art, is not sufficiently balanced by appropriate throughput or sample quality.

### 4.2. Microfluidic Isolation Techniques: Which Is the Most Popular

**Figure 7:**
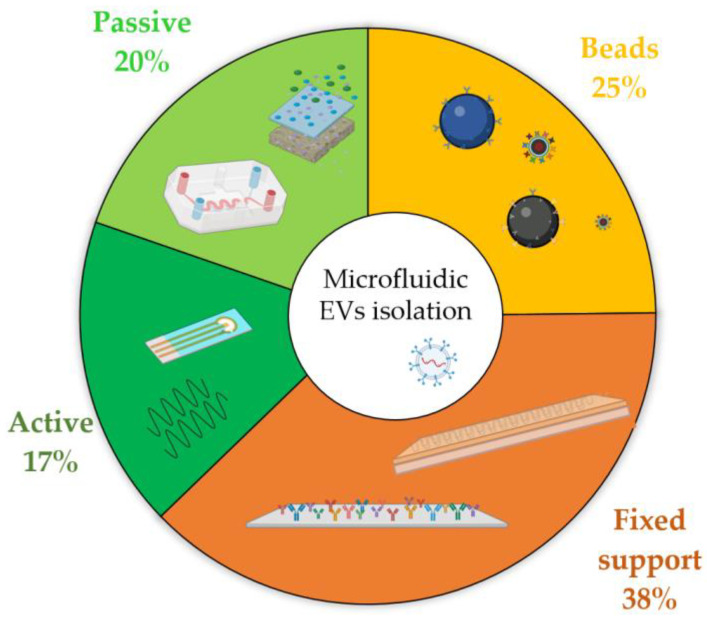
Frequency of the published works from 2000 to present (source: Web of Science database, excluding reviews, meeting abstracts, and retracted papers) related to each of the different isolation techniques that exploit microfluidic devices. Physical approaches are subdivided into active (dark green) and passive (green), whereas chemical immunocapture is separated by fixed supports (orange) and floating beads (yellow).

To better appreciate how widespread these methods are, Figure 7 reports a statistical representation of all the published articles on EV isolation performed by microfluidic devices.

The pie chart in Figure 7 highlights that the predominance of microfluidic methods makes use of chemical immunocapture, which comprises two-thirds of the total output. This is probably due to the fact that physical approaches leverage concepts similar to conventional ones, such as DU, UF, or SEC, hence carrying similar drawbacks in their usage, and automation has not yet been implemented in the presented proof-of-concept device. Moreover, chemical affinity provides an easy implementation inside microfluidic devices, especially for flat channels that require a simple functionalization capable of binding to membrane proteins, such as tetraspanins. Additionally, these methods generally perform better than their physical counterparts, mainly in terms of throughput and recovery rate. Among chemical isolation devices, those exploiting fixed substrates are the most commonly used, probably due to the large amount of literature related to the functionalization of microfluidic devices [192], the automatization capability, and the simple microfabrication, despite the fact that the production of certain integrated nano-structures could lead to some difficulties. In contrast, there is no preferred physical approach for EV purification: passive devices are slightly more frequently used, despite the poor quality of final samples, probably because the users do not need specific training to control SAW and DEP, and these devices are more prone to miniaturization. However, these active approaches are relatively newer with respect to their passive counterparts, and therefore, further development is expected in the future to facilitate device production and handling.

### 4.3. Are Microfluidic Devices for EV Isolation Ready for Clinical Applications

**Figure 8:**
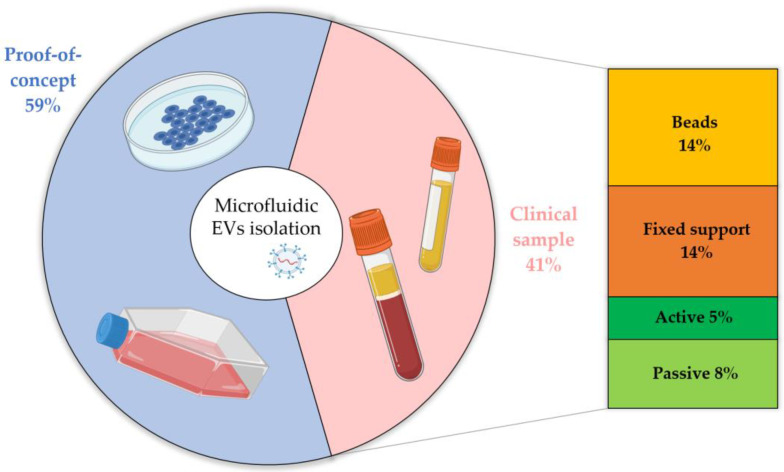
Frequency of published works from 2000 to present (source: Web of Science database, excluding reviews, meeting abstracts, and retracted papers) involving EV isolation from starting biofluids used as a *proof of concept* (cell culture media or EVs spiked in body fluids) or as *clinical samples *(body fluids directly employed) from healthy donors or patients for research studies or diagnostic purposes, further divided according to the isolation technique.

Today, the clinicaltrials.gov database reports more than 350 trials (about 90 already completed) indexed by the keywords *exosomes* and *extracellular vesicles*; among them, only two, which have just started, involve microfluidic systems. The latter can be understood considering the still young character of the EV research field. However, to better analyze the current state of the art from a clinical perspective, we discuss the applicability of the presented microfluidic devices in real diagnostic conditions. In detail, the microfluidic devices devoted to purified and isolated EVs are validated using different starting samples (i.e.,: culture media, blood-derived fluids, urine, etc.), and, in some experiments, EVs are spiked in human fluids. Although we consider all these approaches to be fundamental for the validation of novel technologies, they represent a first proof of concept compared to real clinical assays, since EV isolation may depend on several factors, such as starting samples and EV biogenesis. Therefore, we divided the presented papers based on their validation methods, labeled as: (i) *proof of concept*, if they are limited to cell culture media or spiked EVs in body fluids, and (ii) *clinical sample*, when they directly employ body fluids for EV isolation (e.g., urine, plasma, serum, or whole blood). The result of this classification is reported in Table 4 and Figure 8, together with the isolation approach used. 

In Figure 8, it can be observed that 40% of microfluidic devices have been validated using body fluids taken from healthy donors or real patients, representing a good percentage of the total microfluidics-based studies. Among these works, most use immunoaffinity-based devices to capture vesicles, increasing the difference already noted in Figure 7 compared to the use of physical methods. Notably, passive physical methods are disposable and more prone to clogging than their non-microfluidic counterparts, whereas active interactions in real fluids must consider several parameters, such as the fact that viscous biofluids are not as easy to be manipulated as aqueous solution. It is worth noting that most of the works processing *proof-of-concept* samples, such as cell lines or spiked EVs, also include pre-purification steps (e.g., differential ultracentrifugation) before the injection of the biological fluids to favor better isolation of small vesicles after the elimination of heavier debris and cell fragments. In contrast, a device already tested with clinical samples without requiring pre-treatment can be considered ready to use for medical diagnostics, and this kind of validation must be the final goal of future studies.

### 4.4. Microfluidic Devices for EV Detection and Analysis

Extracellular vesicles collected and purified by the aforementioned approaches can be investigated by several detection techniques. The more conventional ones for EV size estimation require off-chip treatment and characterization by commercial instruments, mostly based on tracking such as nanoparticle tracking analysis (NTA) [205], dynamic light scattering (DLS) [206], or flow cytometry (FC) [207], which reveals information on EV morphology. Indeed, NTA is actually performed in a microfluidic chip for the estimation of size by scattered light based on Brownian particle fluctuations and similarly occurs for DLS; cytometric analysis requires hydrodynamic vesicle focusing that is otherwise achievable by the microfluidic channel. 

Microfluidic devices have also been developed for EV detection, mainly based on two approaches: (i) self-standing, in which the device itself works as detector, and (ii) storage, in which the microchannels act as a storing chamber for EVs that are screened by an external microscope or probe. The first approach typically involves the integration of a devoted electrode, as occurs for analysis by field-effect transistors [185], electrochemical sensing [208], zeta potential estimation [102], or lateral flow immunoassay [172]. 

In contrast, in the second approach, the microfluidic device allows for several types of detection: (i) colorimetric for EV quantification [109], (ii) spectroscopy based on surface-enhanced Raman scattering (SERS) [94], (iii) optical properties by surface plasmon resonance (SPR) [200], (iv) resistive pulse sensing due to EV through nanopores [209], (v) detection by micro-nuclear magnetic resonance (NMR) [142], etc. Furthermore, some microfluidic platforms were engineered to also perform EV content analysis, such as on-chip quantitative PCR [181] or ELISA [44]. More details on detection methods based on microfluidic devices can be found in other specific reviews [20,210].

## 5. Conclusions and Perspective

This review looks at the most important techniques currently used to isolate EVs using microfluidic devices, analyzing their ability to be applied in clinics. In this perspective, despite the fact that microfluidic technologies have only recently been applied to the isolation of EVs, almost half (40%) of the presented devices use potentially clinical samples for their validation. Therefore, microfluidics seems to represent a promising strategy for medical investigation. 

The comparison between physical and chemical microfluidic approaches for EV isolation emphasizes a slight preference for chemical methods over their physical counterparts, which becomes much more evident if one considers only the technologies validated with potential clinical samples. This might be the cause for two possible reasons: (i) the device performance itself (higher throughput, better capture efficiency, and simple microfabrication for mass production) or (ii) the general trend of the biomedical community to look for EVs that have specific phenotypes. Actually, we believe that the latter is dominant, even though there is probably a combination of both factors. Although the characterization of EVs was initially based on quantification and size classification, it is now clear that the specific EV subpopulations can provide useful information related to specific diseases [8]. Therefore, we expect that the gap between physical and chemical approaches will increase in the near future. A possible alternative to further improve the efficiency and purity in EV capture might be the implementation of both physical and chemical approaches combined together inside a single device, using passive EV separation from other massive particles, and then a specific immunocapture for distinguishing different subpopulations, providing also high throughput isolation.

In addition to EV isolation, microfluidic devices are also widely employed for EV detection and analysis. Whereas the first devices consisted of simple microchannels that aimed to store EVs for further analysis (as for the NTA), new approaches, which combine microfluidic systems, devoted optical systems, and antibody immunocapturing, point towards the analysis of single vesicles in order to achieve information about their heterogeneity within the same population [211].

In conclusion, we are confident that microfluidics, which is already employed in some gold-standard techniques for EVs analysis (i.e.,: NTA), can bring great support in terms of EV isolation by providing tools capable of performing repeatable and automated protocols, similar to other important biomarkers, such as CTCs and ctDNA. However, microfluidics experts exposed to the field for the first time must also consider biological and medical points of view. Therefore, to be really useful, new technologies must be developed in accordance with the requirements and expectations that, importantly, have progressively changed over the past five years, following the guidelines on the validation of the protocol and the outcomes established by the ISEV community [9]. 

## Figures and Tables

**Table 1:** Summary of the comparison of conventional techniques for EV isolation, based on different isolation strategies. Retention time, quality, and quantity of processed sample and protocol simplicity are evaluated according to the following scale: (--) difficult/very bad, (-) non-trivial/mediocre, (+) easy/good, (++) very easy/excellent.

	Working Principle	Retention Time	Output Sample (Quality and Quantity)	Simplicity
Differential ultracentrifugation	particle size	-	-	++
Density gradient ultracentrifugation	particle density	--	--	++
Ultrafiltration	particle size	--	-	+
Size-exclusion chromatography	particle size	--	-	+
Field-flow fractionation	particle size	-	+	+
Precipitation-based	particle–polymer interaction	-	+	+
Immunoaffinity-based	antigen–antibody binding	-	+	+

**Table 2:** Summary of relevant published articles dealing with EV isolation from different microfluidic approaches.

	Methods	Working Principle
Physical: Passive	Filtration [38,39,40,41,42,43,44,45,150,151]	Micro-/nano- filtration process by porous membranes inside chip
	Deterministic lateral displacement [46,60,61,62]	Particle distribution in size by lateral forces conveyed by ordered array of posts
	Inertial force [50,51,52,53,54,55,77] Viscoelastic force [67,68,69,70,71,72]	Imbalance of inertial forces or of shear forces in non-Newtonian viscoelastic fluid
Physical: Active	Acoustofluidics [86,87,88,89,90,91,92,93,94,95,96,107]	Acoustic trapping by ultrasound waves
	Electrokinetic force [98,99,100,101,102,103,104,105,106,107,152,153,154]	Charge separation by electric fields
Chemical: Fixed support	Functionalized fixed support [108,109,110,111,112,113,114,115,116,117,118,119,120,121,122,130,155,156,157,158,159,160,161,162,163,164,165,166,167,168,169,170,171,172,173,174,175,176,177,178,179,180]	EV capture by specific antibodies on fixed substrate
Chemical: Floating Beads	Magnetic beads [125,126,127,128,129,130,131,132,133,134,135,181,182,183,184,185,186]	EV capture by specific antibodies on beads for magnetic manipulation
	Polystyrene beads [80,99,124,137,139,140,141,187]	EV capture by specific antibodies on non-magnetic beads
	Magnetic nanoparticles [93,134,138,142,143,144,145,146,147,149,188]	EV capture or handling by specific antibodies on magnetic nanoparticles

**Table 3:** Summary of the comparison of different microfluidic techniques for EV isolation. Each of the four aspects is evaluated according to the following scale: (--) difficult/very bad, (-) non-trivial/mediocre, (+) easy/good, (++) very easy/excellent.

	Throughput	Output Sample (Quality and Quantity)	Possible Automation	Micro- Fabrication Simplicity
Physical: Passive	++	-	+	-
Physical: Active	-	+	+	-
Chemical: Fixed support	+	++	++	+
Chemical: Floating Beads	+	++	--	+

**Table 4:** Summary of articles published during the years 2000–2022 dealing with EV isolation from microfluidic devices processing samples for research purposes or clinical trials, according to techniques classified in Table 2.

	Proof of Concept		Clinical Sample	
	Starting Sample	%	Starting Sample	%
Physical: Passive	Plasma [52,62] Urine [44,60,95] Cell culture [41,42,43,50,51,52,54,55,61,68,71,72,77,88,150,151,193,194]	12	Plasma [39,195] Serum [43,46,71] Blood [45,53,67,69] Urine [38,39,44,46]	8
Physical: Active	Plasma [98,102,103,106,152] Serum [103] Saliva [88] Cell culture [86,89,91,98,99,100,104,105,107,153,154]	12	Plasma [87,91,94] Blood [90,101] Urine [91,92,95]	5
Chemical: Fixed support	Plasma [87,173] Serum [122,168] Cell culture [109,113,122,155,156,157,158,160,161,162,164,165,166,167,172,174,175,176,177,179,180,196,197,198,199,200]	24	Plasma [108,114,119,120,123,155,163,171,173,201] Serum [110,117,118,123,130,178,202,203] Blood [170] Urine [115,159,169]	14
Chemical: Floating beads	Plasma [130] Serum [130,183] Cell culture [99,137,139,140,141,143,147,182,186,188,193,204]	11	Plasma [80,124,125,126,127,129,132,135,138,144,185] Serum [131,134,145,147,148,181] Blood [133,142,146] Urine [128]	14
TOTAL		59		41

## Data Availability

Not applicable.
